# Profiling oral health status, values, and related quality of life in patients with oral cancer: a pilot study

**DOI:** 10.3389/froh.2023.1268657

**Published:** 2023-11-28

**Authors:** Inês Rodrigues, João Botelho, Vanessa Machado, Luís Proença, José João Mendes, Carlos Zagalo

**Affiliations:** ^1^ Egas Moniz Center for Interdisciplinary Research (CiiEM), Egas Moniz School of Health & Science, Almada, Portugal; ^2^ Evidence-Based Hub, Egas Moniz Center for Interdisciplinary Research (CiiEM), Egas Moniz School of Health & Science, Almada, Portugal

**Keywords:** oral cancer, oral squamous cell carcinoma, quality of life, oral health, periodontitis

## Abstract

Patients diagnosed with oral cancer frequently exhibit an increased likelihood of experiencing common dental conditions, lower dental health literacy, and a decline in their overall quality of life. However, there is limited understanding on the impact of oral health values on these patients. This study aims to explore the oral health status and their oral health determinants and reported outcomes in patients with oral squamous cell carcinoma (OSCC) at the *Instituto Português de Oncologia de Lisboa Francisco Gentil*. This cross-sectional study included patients who were diagnosed with OSCC for the first time. These individuals were administered a questionnaire that collected sociodemographic data, as well as their scores on the oral health value scale (OHVS) and the short-form oral health impact profile. In addition, participants self-reported their experiences with periodontitis and caries using the decayed, missing, filled index. The final sample consisted of 46 OSCC patients, consisting of 34 males and 12 females. The average age of the participants was 70.0 years (±13.2), with most of them being retired (65.2%). There were no differences found between sex regarding age (*p* = 0.531), employment status (*p* = 0.114), presence of systemic conditions, smoking habits (*p* = 0.423), or alcohol consumption (*p* = 0.404). In the OHVS, there was a statistically significant difference between men and women regarding the retention of natural teeth (*p* = 0.021). Patients with self-perceived periodontitis exhibited worse functional limitation (*p* = 0.039) and physical pain (*p* = 0.049). Regarding dental caries experience, it was observed that a majority of patients had a higher incidence of missing posterior teeth (59.2%). This sample presented a significant decline in oral health, in addition to the diagnosed OSCC. The oral health value and quality of life were severely declined. There is a concerning lack of oral care and health that, consequently, impacts the quality of life of these patients.

## 1. Introduction

Oral cancer is among the 10 most prevalent types of cancer worldwide (1). It is estimated to represent 2% of all malignant tumours globally, with 377,000 new cases and 177,000 deaths annually (2). Particularly, oral squamous cell carcinoma (OSCC) is the predominant form of oral cancer, with the tongue being the most frequent site in the oral cavity (3). The condition is characterised by the presence of an ulcerated lesion. It is asymptomatic in an early stage. However, with the progression of the disease, the affected individuals may experience pain and dysphagia, which can be attributed to the invasion of the surrounding structures (3).

When diagnosed, curative treatment for oral cancer can start with surgery and then be followed by chemotherapy and radiotherapy, depending on its stage (4). Consequently, the masticatory function, ability to speak, aesthetic aspects, and oral somatosensory perception (taste and smell) are compromised, leading to a decrease in the quality of life (4, 5). In addition, oral complications such as xerostomia, mucositis, trismus, and increased risk of infectious diseases are common during the curative treatment (6).

Periodontitis is a chronic inflammatory disease that is induced by the presence of bacteria in the biofilm of the oral cavity. This disease leads to bone resorption, loss of periodontal support, and loss of dental pieces (7–9). In the last decade (2011–2020), it was estimated that the prevalence rate of periodontitis in adults was approximately 62%, and the prevalence rate of its severe form was 25% (10). This disease not only has a negative impact on function, but also on general health (8). Periodontitis is also linked to some systemic diseases such as diabetes, cardiovascular diseases, and adverse pregnancy outcomes (11–13). Nevertheless, the association between periodontitis with head and neck cancer has been recently reported (14, 15).

Dental decay is the most prevalent oral disease in the world, and it is characterised by a demineralisation process caused by bacteria present in the oral biofilm (16). When left untreated, symptoms such as discomfort, pain, and infections may arise, which can ultimately result in the loss of teeth (16, 17). Consequently, it is expected that the affected individuals may experience difficulties in chewing and speaking (16, 18, 19).

The loss of dental pieces reflects one of the most serious consequences of untreated diseases such as caries and periodontal disease, and it is frequently observed among the elderly population (17, 20, 21).

There is no doubt that the quality of life of people with these oral diseases is compromised, not only due to function limitation but also due to decreased aesthetics, low self-esteem, and negative impact of the psychological state.

Therefore, this study aims to report the oral health status, the perception of the quality of life of patients diagnosed with OSCC, and the medical and sociodemographic data at the *Instituto Português de Oncologia de Lisboa Francisco Gentil* (IPOLFG). Ultimately, the results of this study will provide a snapshot of the overall condition of these patients and set the tone for the importance of oral health among oncology healthcare workers and institutions.

## 2. Materials and methods

### 2.1. Study design, setting, and participants

This cross-sectional study was developed at the *Instituto Português de Oncologia de Lisboa Francisco Gentil*. It received approval from the respective Institutional Review Board (Ethics Committee of IPOLFG, ID: 1539) on 5 January 2023, and adhered to the guidelines outlined in the Declaration of Helsinki of 1975, as revised in 2013. This study is reported in accordance with the Strengthening the Reporting of Observational Studies in Epidemiology (STROBE) statement for observational studies (22). The participants who met the inclusion criteria were first-arriving patients with a diagnosis of OSCC at IPOLFG; patients who were able to understand and sign the informed consent form or have a legal representative authorising them to do so; and patients diagnosed with an OSCC and underwent a tumour biopsy, which was analysed by an anatomopathologist, who confirmed the diagnosis of an OSCC (3). Patients were excluded if they were already under treatment (chemo- and/or radiotherapy) for OSCC. The study employed a non-probabilistic consecutive sampling method to choose the patients, with data collected from January 2023 to May 2023. The data were collected at the Head and Neck Unit at the IPOLFG, where patients completed the questionnaire and underwent clinical observation immediately following their scheduled appointment.

### 2.2. Variables

#### 2.2.1. Sociodemographic questionnaire

We collected information regarding age, sex, occupation status (employed, unemployed, or retired), medical conditions, smoking habits (never, former, or current smoker) and number of cigarettes per day, and alcohol consumption (active or passive).

#### 2.2.2. Oral health values scale

To measure the value that a person places on his/her own oral health (23), we employed the Portuguese version of the oral health value scale (OHVS-PT) (24). This 12-item tool assesses relevant OHV domains, namely, professional dental care (items 4, 8, and 11), appearance and health (items 3, 7, and 12), flossing (items 2, 5, and 10), and retaining natural teeth (items 1, 6, and 9). Each question is rated on a five-point scale: 1 = “Strongly disagree,” 2 = “Disagree,” 3 = “Neutral,” 4 = “Agree,” and 5 = “Strongly agree” (23). The final overall score is calculated as per recommendations for scale construction (23) by summing up all items and reverse-scoring items 2, 4, 6, 8, 9, and 11. To allow a proportional assessment of OHVS, we then converted the values to percentages.

#### 2.2.3. Oral health-related quality of life

The oral health-related quality of life (OHRQoL) was measured using the short-form oral health impact profile (OHIP-14) validated for Portuguese (25). OHIP-14 assesses 14 items, each of the items rated on a five-point ordinal scale (“Never ” = 0, “Hardly ever” = 1, “Occasionally ” = 2, “Fairly often ” = 3, and “Very often” = 4). We then obtained the seven domains of OHIP-14: “Functional Limitation” (items 1 and 2), “Physical pain” (items 3 and 4), “Psychological discomfort” (items 5 and 6), “Physical disability” (items 7 and 8), “Psychological disability” (items 9 and 10), “Social disability” (items 11 and 12), and “Handicap” (items 13 and 14). In addition, we further accounted for three super domains entitled “OHIP-14 Physical” (items 1, 2, 3, 4, 5, and 10 were summed), “OHIP-14 Psychological” (items 6, 7, 8, and 9 were summed), and “OHIP-14 Social” (items 11, 12, 13, and 14 were summed) (25).

#### 2.2.4. Self-report measures of periodontitis

Considering the setting in IPOLFG, we employed a surveillance strategy based on a self-reported questionnaire for periodontitis, previously validated in Portugal. This questionnaire accounts for three questions with a dichotomous response (Yes vs. No). It is a self-report questionnaire in order to recognise the presence of “Gum disease,” “Bone loss,” “Gum treatment,” “Loose teeth,” since they were significant questions regarding risk factors (26). The questionnaire consists of 13 questions in Portuguese: (1) Do you think you might have gum disease?; (2) Overall, how would you rate the health of your teeth and gums; (3) Have you ever had treatment for gum disease, such as scaling and root planning, sometimes called “deep” cleaning?; (4) Have you ever had any teeth become loose on their own, without an injury; (5) Have you ever been told by a dental professional that you lost bone around your teeth?; (6) During the past 3 months, have you noticed a tooth that doesn't look right?; (7) Aside from brushing your teeth with toothbrush, in the last 7 days, how many times did you use dental floss or any other device to clean between your teeth?; (8) Do your gums usually bleed either when brushing or chewing?; (9) During the past 3 months, have you had bleeding gums?; (10) Have you lost teeth in recent years because of mobility?; (11) Have you felt pain in your gums during the last months?; (12) In the past years, have you noticed that your teeth are longer or that you have receding gums?; (13) In the last years, have you noticed that you see the roots of several of your teeth? (26).

#### 2.2.5. Dental caries experience

In order to report the presence of dental caries and lost teeth, the decayed, missing, filled (DMF) index was used. The DMF index is a measure of dental status that describes past and present caries experience (27). The decayed, lost, and filled teeth were registered through clinical observation, by the investigator who was collecting the data at the end of the consult at IPOLFG, with the patient seated in the examination chair available in the room. As such, the following value was attributed to each tooth as follows: “D” (decayed), a tooth with visible signs of decay, such as cavities, softening, or discolouration; “M” (missing), a tooth that was missing for any reason; and “F” (filled), a tooth that has a dental filling or restoration without signs of decay.

### 2.3. Sources of bias

According to the nature of this study, the sources of bias are plausible to occur. Variables such as OHVS, OHRQoL, and self-report measures of periodontitis were evaluated through a questionnaire that can lead to response bias. The completion of the questionnaire was always supervised by the same investigator to prevent response bias (28).

The data collected for the DMF index were registered by the same investigator to minimise other potential sources of bias.

### 2.4. Statistical analysis

We performed data normality tests to assess the normality of the continuous variables within our dataset. We used the Shapiro–Wilk test to assess the adherence of the data to a normal distribution. After verifying the absence of a normal distribution, we proceeded to employ non-parametric tests for comparison of continuous and categorical variables.

To compare continuous variables between groups or categories, the Mann–Whitney *U* test was employed for independent samples to compare two groups, and the Kruskal–Wallis test to compare several groups. These tests assess whether there are significant differences in the medians of the variables between groups or categories.

For categorical variables, we used Fisher's exact test. These tests allow us to determine whether there are significant associations or differences between categorical variables.

We used the ggplot2 package of R for data visualisation. Specifically, we used boxplots to visualise the distribution of continuous variables across different groups or categories. Boxplots provide a graphical representation of the median, quartiles, and any potential outliers in the data, allowing for a quick assessment of the overall distribution and comparison between groups. We also used circle plots (also known as circular bar charts or polar bar charts) to present categorical variables. The pie charts provide a circular representation of categorical data, allowing for a visual exploration of proportions or frequencies in a circular format. Continuous values are presented as means and standard deviations, and categorical values are presented as percentages. The level of significance was set to *p* < 0.05.

## 3. Results

### 3.1. Characteristics of the study sample

**Figure 1:**
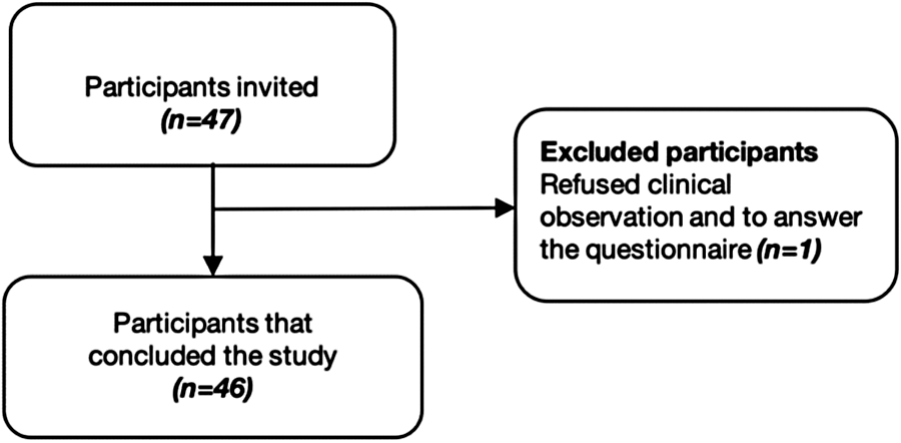
Flowchart of patient inclusion. The data describe the steps involved in the inclusion of the final sample with the number of patients and respective reasons.

From an initial sample of 47 participants, one person refused to answer the questionnaire and clinical observation (Figure 1). The final 318 sample consisted of 46 patients with a confirmed diagnosis of SCC, 319 with a predominance of men (34 males vs. 12 females) (Table 1). The participants had an average age of 70.0 years (±13.2), most of them were retired (65.2%). We found no differences between men and women regarding age (*p* = 0.531), employment status (*p* = 0.114), presence of systemic conditions, smoking habits (*p* = 0.423), or alcohol consumption (*p* = 0.404). The most common systemic disease was hypertension (45.7%). Considering the average number of newly diagnosed SSC cases per year in Portugal in 2021, the present sample of participants represents 4.2% of the total cases reported at the national level, and 13.1% of all cases observed at the IPOLFG.

**Table 1.** Sociodemographic characteristics of the study sample, stratified by sex (*n* = 46).

	Male (*n* = 34)	Female (*n* = 12)	*P*-value	Total (*n* = 46)
Age (years), mean (SD)	71.5 (14.0)	68.1 (13.0)	0.531	70.0 (13.2)
Employment status, n (%)				
Employed	9 (26.5)	3 (25.0)	0.114	12 (26.1)
Unemployed	3 (8.8)	1 (8.3)		4 (8.7)
Retired	22 (64.7)	8 (66.7)		30 (65.2)
Systemic Diseases, n (%)				
None	9 (26.5)	5 (41.7)	0.536	14 (30.4)
At least one systemic condition	25 (73.5)	7 (58.3)	0.489	32 (69.6)
Diabetes mellitus	6 (17.6)	0 (0.0)	0.154	6 (13.0)
Hypertension	18 (52.9)	3 (25.0)	0.282	21 (45.7)
Cardiovascular Disease	12 (35.3)	2 (16.7)	0.361	14 (30.4)
DPOC	1 (2.9)	0 (0.0)	0.554	1 (2.2)
Liver Transplant	2 (5.9)	0 (0.0)	0.404	2 (4.3)
Rheumatoid arthritis	1 (2.9)	0 (0.0)	0.554	1 (2.2)
Stroke	3 (8.8)	0 (0.0)	0.309	3 (6.5)
Multiple Sclerosis	0 (0.0)	1 (8,33%)	0.102	1 (2.2)
Breast cancer	0 (0.0)	2 (16.7)	0.024*	2 (4.3)
Prostate Cancer	4 (11.8)	0 (0.0)	0.241	4 (8.7)
Colon Cancer	2 (5.9)	0 (0.0)	0.404	2 (4.3)
Smoking habits, n (%)				
Former	11 (32.4)	2 (16.7)	0.423	13 (28.3)
Active	11 (32.4)	1 (8.3)		12 (26.1)
Never	12 (36.4)	9 (75.0)		21 (46.7)
Alcoholic drinks per day, n (%)				
1 time or more	22 (64.7)	2 (16.7)	0.404	21 (45.7)
<1	4 (11.8)	2 (16.7)		2 (4.3)
None	8 (23.5)	8 (66.6)		16 (34.7)
Periodontitis self-report, n (%)				
Yes	17 (50.0)	3 (25.0)	0.323	20 (43.4)

### 3.2. Oral health value

Regarding Table 2, there is a statistically significant difference in retaining natural teeth (*p *= 0.021) between males and females. Nevertheless, there is a remarkable lack of flossing in both sexes (18.8%).

**Table 2.** OHVS and each domain, stratified by sex (*n* = 46).

OHVS, mean (SD) (%)	Male (*n* = 34)	Female (*n* = 12)	*P*-value	Total (*n* = 46)
Total	54.1 (12.8)	64.1 (17.8)	0.071	56.7 (14.7)
Professional Dental Care	65.3 (18.9)	61.3 (19.4)	0.324	62.3 (18.9)
Appearance and Health	85.0 (14.2)	90.3 (15.8)	0.162	86.4 (14.6)
Flossing	15.0 (19.7)	29.9 (31.5)	0.114	18.8 (23.9)
Retaining Natural Teeth	55.1 (17.5)	70.8 (22.6)	0.021*	59.2 (20.0)

SD, Standard deviation

### 3.3. OHRQoL

As for the OHRQoL, no statistically significant differences were found between men and women. However, more than a half of the sample (51.6%) were psychologically affected.

#### 3.3.1. OHRQoL and self-report measures of periodontitis

Comparing the self-perception of periodontitis with OHIP-14 domains, there were statistically significant findings observed concerning functional limitation (*p* = 0.039) and physical pain (*p* = 0.049).

### 3.4. Dental caries experience

The posterior teeth, specifically teeth 16 and 26, had the highest prevalence of caries, while tooth 45 and the superior anterior teeth, teeth 11 and 21, demonstrated the lowest incidence of caries. On the other hand, tooth 21 exhibited the highest frequency of loss within the anterior sector, whereas teeth 16, 26, 37, 36, 35, 46, and 47 were the most frequently lost teeth in the posterior sector. Regarding filled teeth, it is noteworthy that only teeth 47 and 34 exhibit no evidence of restorative treatment in the posterior region, but then teeth 15, 14, 35, and 26 demonstrate the highest prevalence of restoration. The anterior sector was the least affected by dental decay, restorative treatment, and tooth loss.

Based on the findings shown in Table 3, it is evident that the posterior teeth, particularly the molars, exhibit the highest prevalence of tooth loss (59.2%) and decay (13.8%), while the canines have the lowest rate of tooth loss (32.0%) and the most unaffected (61.9%) ones. Approximately 50.6% of the lost teeth were located in the first quadrant. Regarding the lower teeth, the inferior teeth have the lowest degree of dental restorations (1.7%).

**Table 3.** Distribution of unaffected and each domain of DMF index per maxilla, quadrant and tooth tupe (*n* = 46).

Teeth	Unaffected	D	M	F
Upper	39.0	8.7	49.5	2.8
Lower	45.3	8.5	44.4	1.7
Quadrants				
1	37.9	8.4	50.6	3.1
2	40.1	9.0	48.4	2.5
3	45.0	8.1	45.0	1.9
4	45.7	9.0	43.8	1.6
Tooth type				
Anterior	54.3	4.3	41.3	0.0
Canine	61.9	5.9	32.0	0.0
Premolar	33.4	8.9	47.8	4.0
Molar	23.1	13.8	59.2	3.7

## 4. Discussion

### 4.1. Main results

The main aim of this pilot study is to report the oral health status and oral health-related quality of life valuation of patients with a first diagnosis of an OSCC, who attended a head and neck consult at the IPOLFG.

On the overall, the oral health of the sample population exhibited significant deterioration, with approximately 21.7% being totally edentulous and 43.7% reporting the presence of periodontitis. Consequently, it was expected that the study population was psychologically affected (51.6%). Surprisingly, people with a self-report of periodontitis had both functional (*p* = 0.039) and physical limitations (*p* = 0.049).

This study also aims to raise awareness of the importance of oral health within the healthcare community, particularly among health professionals involved in the treatment of cancer patients, to mitigate the repercussions that such treatments may have on oral health.

On the other hand, being able to create an oral health profile of patients with head and neck cancer not only allows us to identify potential oral diseases that may be associated with underlying pathological conditions, but also provides insight into the state of oral health in Portugal and the difficulties and needs in terms of oral care.

The results showed that the population under study has its own characteristics, which were found to be consistent between men and women.

With regard to the OHVS, one of the parameters that merits attention is the relatively low prevalence of flossing, which is reported to be practised by only 18.8% of the population. In contrast, when it comes to the appearance and health domain, the results showed a high valuation (86.4%), so the study population recognises that oral health is an important factor not only in terms of their appearance but also acknowledges its importance in general health. This leads us to believe that although it is considered an integral factor in the individual's wellbeing, there are barriers such as lack of accessibility, lack of incentive, and lack of literacy that makes it a challenge to achieve, since flossing, an important step of the oral hygiene, is depreciated. When it comes to retaining natural teeth, women have a higher valuation (70.8%) comparing with men (55.1%), and a statistically significant difference was found, in this domain, between sexes (*p* = 0.021). This leads us to believe that women have a higher self-care than the opposite sex when it comes to their natural dentition, and it can be related to the fact that they are more worried about the physical aesthetics and have a higher valuation of their oral health (64.1%).

The quality of life of the study sample, measured by the OHIP-14, was severely compromised, with 51.6% being psychologically affected. There are several factors that may be related to these results such as poor oral health, the absence of dental pieces, and the presence of caries. These not only compromise chewing and speaking abilities, but also have a strong impact on self-esteem (19, 29). On the other hand, the presence of a tumour in the oral cavity can essentially limit function and aesthetics depending on its size (3). In addition, the perception of the existence of the tumour and the whole process up to the determination of a definitive diagnosis causes considerable anxiety, compromising the patient's psychological status. This outcome is extremely concerning from a prospective standpoint, as these patients will undergo aggressive treatments that will have great repercussions on their oral cavity, posing considerable challenges to deal with. The multidisciplinary team must prioritise the maintenance of the patients’ quality of life, since it is crucial for their motivation and overall wellbeing.

Patients who self-reported periodontitis (43.4%) had functional impairments (*p* = 0.039) and experienced physical pain (*p* = 0.049). These data are of significant concern due to their association with individuals diagnosed with OSCC among the studied group. Periodontitis not only compromises the aesthetics but also the function, making it difficult to chew, talk, and socialise (8). The quality of life of this study sample is already affected by the presence of a malignant tumour in the oral cavity that, depending on its stage and size, compromises function. Hence, in the presence of periodontitis, a greater decline on the quality of life is expected. In addition, periodontitis is a chronic inflammatory disease, so an adequate treatment must be required to prevent and attenuate any outcomes of curative treatments.

The DMF index revealed a substantial loss of molars (59.2%), the teeth essential for chewing. Regarding anterior teeth, 41.3% were lost due to caries, which inevitably affects aesthetics and self-esteem and compromises the patient's psychological state and quality of life. It is imperative to address any dental decay prior to undergoing chemotherapy or radiotherapy, as this not only serves to prevent the need of extractions, which can lead to more severe complications such as osteonecrosis, but also helps to maintain the individual's natural teeth, hence maintaining their ability to chew and preserving their self-esteem (3).

### 4.2. Strengths and limitations

The observational design and the sample size confer a pilot structure to this study. However, when analysing the representativeness, this portrays 13.1% of all cases seen in the IPOLFG in 2021, which contributes to a greater robustness and confidence of the results.

Furthermore, it is important to consider that the results were obtained from questionnaires, which introduces the possibility of response bias and could influence the quality of the obtained results (28). It is also important to mention that the questionnaires of the self-report measures of periodontitis have been validated and proven to be an effective method for evaluating the prevalence of periodontitis in the population. However, it is crucial to verify the diagnosis of periodontitis through clinical observation (26).

### 4.3. Comparative studies

The results obtained are unique in Portugal. However, similar studies have been conducted in other countries using the same or similar tools to perceive the oral health status of patients with head and neck cancer.

In the United Kingdom, a study was conducted in 2014 with 100 patients with a first diagnosis of head and neck cancer, in which 91% presented an OSCC. The study utilised the DMF index to record the dental caries experience, and periodontal probing was conducted to assess the presence of pockets of 4 mm or greater in depth (30). The results showed that a total of 2% of the participants in the study were fully edentulous, while 71% had periodontitis, and 61% had one or more instances of caries (30). Although a different tool was used in this study in the diagnosis of periodontitis, evidence shows that the self-report questionnaire is equally valid, effective, and practical in the diagnosis of this condition (31).

A study conducted in India in collaboration with the Oxford Dental College Hospital and Research Centre in 2013 assessed 242 patients with OSCC and their oral health (32). In order to assess the past experience of dental decay, they used the DMF index. However, they included third molars. To determine the presence of periodontitis, they used the community periodontal index (CPI). Furthermore, this study distinguished the results in stages of the OSCC (early and advanced stage). The results reported that 2.9% of the participants in the study were edentulous, which is a significantly lower value compared with our results (21.7%). More than half of the teeth were decayed both in individuals with an early-stage lesion (55.8%) and those in advanced stages (58.3%). Regarding periodontitis, they determined a high prevalence rate of periodontal destruction. It is important to emphasise the fact that CPI is only an indicator of the need of periodontal treatment. It does not allow us to distinguish periodontal diseases such as gingivitis and periodontitis. Therefore, it is not possible to assess the prevalence of periodontitis in a community using the CPI, unlike using the self-report questionnaire (33, 34).

## 5. Conclusion

Patients with OSCC had a high prevalence of self-reported periodontitis, dental caries experience, and tooth loss. Along with this, the quality of life and psychological state were diminished. These preliminary results pave the way for urgent oral health prevention and care in patients with OSCC.

## Data Availability

The original contributions presented in the study are included in the article/Supplementary Material, further inquiries can be directed to the corresponding author.
